# When Pandemic Hits: Exercise Frequency and Subjective Well-Being During COVID-19 Pandemic

**DOI:** 10.3389/fpsyg.2020.570567

**Published:** 2020-09-24

**Authors:** Ralf Brand, Sinika Timme, Sanaz Nosrat

**Affiliations:** ^1^Sport and Exercise Psychology, University of Potsdam, Potsdam, Germany; ^2^Department of Kinesiology, Iowa State University, Ames, IA, United States; ^3^Department of Health Sciences, Lehman College/CUNY, New York, NY, United States

**Keywords:** mood, motivation, physical activity, habit, health

## Abstract

The governmental lockdowns related to the COVID-19 pandemic have forced people to change their behavior in many ways including changes in exercise. We used the brief window of global lockdown in the months of March/April/May 2020 as an opportunity to investigate the effects of externally imposed restrictions on exercise-related routines and related changes in subjective well-being. Statistical analyses are based on data from 13,696 respondents in 18 countries using a cross-sectional online survey. A mixed effects modeling approach was used to analyze data. We tested whether exercise frequency before and during the pandemic would influence mood during the pandemic. Additionally, we used the COVID-19 pandemic data to build a prediction model, while controlling for national differences, to estimate changes in exercise frequency during similar future lockdown conditions depending on prelockdown exercise frequency. According to the prediction model, those who rarely exercise before a lockdown tend to increase their exercise frequency during it, and those who are frequent exercisers before a lockdown tend to maintain it. With regards to subjective well-being, the data show that those who exercised almost every day during this pandemic had the best mood, regardless of whether or not they exercised prepandemic. Those who were inactive prepandemic and slightly increased their exercise frequency during the pandemic, reported no change in mood compared to those who remained inactive during the pandemic. Those who reduced their exercise frequency during the pandemic reported worse mood compared to those who maintained or increased their prepandemic exercise frequency. This study suggests that under similar lockdown conditions, about two thirds of those who never or rarely exercise before a lockdown might adopt an exercise behavior or increase their exercise frequency. However, such changes do not always immediately result in improvement in subjective well-being. These results may inform national policies, as well as health behavior and exercise psychology research on the importance of exercise promotion, and prediction of changes in exercise behavior during future pandemics.

## Introduction

Regular exercise and physical activity improve physical fitness and help reduce the incidence of various chronic diseases and physical disabilities (Warburton and Bredin, 2017). Although there are risks of injury associated with participating in certain sports, regular exercise and physical activity are recommended to be part of a healthy lifestyle. The World Health Organization (WHO) advises adults to accumulate at least 150 min of moderate-intensity aerobic physical activity during the week, and additional muscle strengthening activities on two or more days a week (WHO, 2018). These activities should be performed in bouts of at least 10 min in duration during most days of the week.

Exercise also has psychological benefits and is believed to lead to better subjective well-being (SWB). Although many people will understand the meaning of the concept “well-being” intuitively, there are different views about its conceptual makeup. In psychology, SWB is often defined as a multi-faceted construct composed of affective and cognitive components (Diener et al., 1999). Defining these components is important as they have often been used wrongly and interchangeably in the literature (Ekkekakis, 2011). At the core of the affective component is a valenced feeling (pleasure/displeasure) that is primitive and does not require cognitive processing (Russell and Feldman Barrett, 2009). Core affective feelings are always present in emotions and moods. Both emotions and mood imply cognitive appraisal, and a strong cultural influence is assumed on their formation. Emotional states (e.g., fear, guilt, and pride) are often short-lived, higher-intensity responses to identifiable stimuli; whereas, moods (e.g., irritation, cheerfulness, and grumpiness) are often less intense and longer-lived, and sometimes have less identifiable stimuli (Ekkekakis, 2011). There are chronic and acute effects of exercise on SWB. Both have been studied with the general population and in people living with chronic disease.

There are numerous studies on the chronic psychological benefits of exercise, and many of them relate to changes in mood. For example, data from genome-wide association studies with 611,583 adult participants show that physical activity (measured via accelerometry) is a protective factor against the risk of developing Major Depressive Disorder (Choi et al., 2019). A meta-analysis of prospective cohort studies suggests that the protective effects of physical activity against depression are comparable in youth, adults, and the elderly population across the globe (Schuch et al., 2018). The chronic effects of exercise on SWB are similar in healthy individuals (Diener et al., 2017; Panza et al., 2019), although studies in this area are sometimes of lower methodological quality than those related to depression.

Population-based survey studies for example showed that those who exercise at least two to three times a week, report significantly less stress, cynical distrust, and anger than less active individuals (Hassmén et al., 2000). Although the majority of the studies on chronic psychological benefits of exercise confirm that exercise can contribute to better SWB and mood (Wicker and Frick, 2017), and has protective effects against depression (Ekkekakis, 2015; Schuch et al., 2016), it would be wrong to claim that exercise always and automatically leads to more well-being. For example, an epidemiological study including 162 monozygotic twin pairs illustrated that a twin, although he or she claims to be an exerciser, will not necessarily report better well-being than his or her less active sibling (Stubbe et al., 2007). Although genetic factors influence both exercise behavior and well-being (Stubbe et al., 2007; Schutte et al., 2017), the relationship between the two variables is obviously mediated by other variables.

Actually, there are nuances with respect to how people feel with a single bout of exercise. While research findings on the acute effects on SWB and mood are often difficult to interpret, the evidence on the effects on core affect is very clear (Ekkekakis and Brand, 2019). During exercise, intensity is a moderating variable of eventual “feel better” effects. Positive affect is most likely to appear with low- and only sometimes with moderate-intensity exercise. During vigorous-intensity exercise negative changes in core affective valence become universal due to the dominance of aversive proprioceptive sensations (e.g., heavy breathing, intense sweating, and sore muscles) (Ekkekakis et al., 2011). Learned cognitive appraisals are needed to transform perceived negative core affect during and after exercise into a resulting overall state of positive SWB. Once such appraisal has been learned (Antoniewicz and Brand, 2016), exercise might begin to have its beneficial effects on mental health. Therefore, in order to benefit from these positive changes, exercise has to be performed regularly and consistently over a period of time.

According to a recent literature review initiated by the United Kingdom Economic and Social Research Council, exercise-induced improvements in well-being may even be considered as an effective measure to increase labor productivity on a national scale (Isham et al., 2020). Against the background of all these findings, it is not surprising that international health organizations and national governments have committed themselves to facilitating and promoting exercise for the public (Breda et al., 2018).

In the early months of 2020, COVID-19 pandemic reached a peak in many countries and by March and April, almost all countries around the world reinforced a certain type of lockdown restriction. The restrictions which can affect exercise behavior include closing of gyms and fitness clubs, as well as restricted access to parks and outdoor environments.

This brief period of time provided a unique opportunity to investigate the effects of externally imposed restrictions on exercise behavior and the respective changes in SWB (mood) on a very large and global scale. We expected to see an overall decrease in exercise levels with respective negative effects on mood. The study further aimed to establish a prediction model that can estimate the changes in exercise behavior during a lockdown depending on prepandemic exercise behavior. A model like this may be useful to predict changes in the future for conditions similar to the governmental lockdowns in the early months of 2020.

## Materials and Methods

This study used a cross-sectional design to investigate changes in exercise behavior during the lockdown restrictions of the current coronavirus pandemic and its relation to changes in mood. We used the brief period of lockdown restrictions and tried to reach participants all over the world. A mixed (fixed and random) effects model approach was used to analyze data. The study was conducted by the International Research Group on COVID and exercise (IRG). Data were collected between March 29, 2020 and May 7, 2020. The IRG is headed by the three authors of this article, and consists of 34 researchers who helped to make the questionnaire available in 18 languages (Arabic, simplified and traditional Chinese, English, Farsi, Filipino, Finnish, French, German, Greek, Icelandic, Italian, Malayan, Polish, Portuguese, Russian, Spanish, and Turkish). All IRG members are listed in the acknowledgments of this article.

### Sample Size

No statistical methods were used to predetermine sample size. IRG members disseminated the link to the survey in their home countries (and possibly beyond) via personal networks, social media and press releases. Because different nations relaxed their COVID-related rules and regulations at different times in March, April, and May 2020, the IRG networkers were responsible to indicate when such changes would impact our research to the extent that would render the research question meaningless (e.g., opening of gyms, outdoor parks, etc.). For example, on April 19, restrictions in Germany were decisively relaxed; therefore, we decided to exclude the subsequent collected data from our statistical analyses. However, the sample size corresponds to the recommendations for sufficient power in hierarchical modeling (Nakagawa et al., 2017).

### Participants

A total of 16,137 individuals from 99 countries filled out the questionnaire. Information on presence of COVID-19 symptoms or a diagnosis was collected to exclude these individuals from the statistical analyses (*n* = 1,085). No further information, for example on physical or mental disabilities, was collected in this study. For the mixed effect models (see below), only data from countries with more than 100 participants were used. The statistical analysis thus included data from participants in Europe, Asia, as well as South and North America. There were five countries with more than 1,000, 11 with more than 500, and 18 with more than 100 participants (countries and exact numbers of participants in countries are listed in Table 1). This resulted in a total sample of 13,696, who were on average 34.1 years old (*SD* = 14.4); men (39.1%), women (59.5%), and participants with other gender identities (1.4%). Of those, 13,673 participants provided full data for the exercise behavior change analysis and 13,500 for the analysis of the associations between exercise behavior and mood. Descriptive statistics on gender distributions and age in countries are summarized in Table 1.

**TABLE 1:** Descriptive statistics on gender and age distribution across different nations.

Country	*n*	% female	Mean age (*SD*)
Austria	146	53	31.8 (±12.3)
Brazil	595	63	34.4 (±11.6)
China	821	55	26.4 (±10.3)
Finland	472	62	41.4 (±11.5)
Germany	2,061	61	37.5 (±13.5)
Greece	156	58	32.5 (±12.8)
Iceland	826	76	41.2 (±12.5)
Iran	206	68	34.0 (±9.6)
Italy	1,834	49	37.0 (±16.3)
Malaysia	379	62	32.2 (±12.5)
Philippines	1,202	58	32.4 (±13.2)
Russia	118	57	24.8 (±10.4)
Spain	592	54	31.3 (±12.5)
Switzerland	2,222	67	29.7 (±12.9)
Taiwan	1,103	57	35.9 (±15.1)
Turkey	680	62	30.4 (±20.1)
United Kingdom	102	59	41.1 (±13.2)
United States	181	70	39.3 (±13)
Analyzed data set	13,696	59	34.1 (±14.4)
Additional participants (not included in the analysis)	2,441	55	36.2 (±14.2)
Total	16,137	59	34.4 (±14.4)

### Variables

The data were collected with an online survey by using the Unipark web-based survey software. Participants were able to skip any questions they did not want to answer or stop answering the questions entirely at any point.

#### Exercise Behavior

Exercise frequency during the pandemic was measured with the question “how often have you exercised lately (during COVID-19)?” Possible answers were “never,” “once in a while,” “once a week,” “2 days a week,” “3 days a week,” “4 days a week,” “5 days a week,” “6 days a week,” and “every day.” We defined exercise for participants as any activity they choose to do as their exercise (e.g., workouts at home, running outside, etc.). Participants were also informed that any physical activity as part of their occupation must not be included unless they are a professional fitness coach or have a similar profession. For statistical analysis, the answers “once in a while” and “once a week” were combined as “1 day or less,” the answers “2 days a week” and “3 days a week” were combined as “2–3 days,” “4 days a week” and “5 days a week” were combined as “4–5 days” and “6 days a week” and “every day” were combined as “almost every day.” Exercise frequency before the pandemic was measured and processed in the same format.

In addition, participants were also asked about their typical exercise intensity (“What would you say the intensity of this exercise was each time you did it?”) and they could respond choosing “low,” “moderate,” “high,” or “very high” intensity. The options “high” and “very high” intensity were combined to “high intensity” for the analysis, because the original distinction did not yield further insights. Participants were also asked about their exercise session length during the pandemic compared to prepandemic (“Were your exercise sessions during COVID-19 on average shorter or longer than before COVID-19?”) and could choose between “shorter,” “longer,” or “they were of about the same duration.”

#### Mood

Mood was measured with 16 items from the Profile of Mood Scale (POMS) (McNair et al., 1971). The POMS is a heavily used psychometric questionnaire that measures general well-being in the clinical field both with the general population and people with chronic disease as well as in sport and exercise psychology research (Leunes and Burger, 2000). In its original form it presents a list of 65 adjectives that describe feelings people have (e.g., “tense” and “active”). The participants are asked to rate each item (adjective) by indicating whether they experienced the respective feeling “not at all,” “a little,” “moderately,” “quite a lot” or “extremely” now and/or in the past few days. In our study, participants were asked to report how they felt “in the last few days during COVID-19.”

The POMS exists in various short versions and language formats, each with different combinations of items which results in different subscales. It is often impossible to match the items from the translated version to the English version with certainty, perhaps due to historic reasons and that there were different standards in transparency and reproducibility of research 20–40 years ago. For this study, we used the 16-item POMS from a German short screening version, which was psychometrically tested using data from a large national and representative sample (Petrowski et al., 2020). The German items were then matched with the English originals by the authors as thoroughly as possible, and translated from English into the respective survey language by the IRG networkers. These 16 items allow subscores for “depression/anxiety,” “vigor,” “fatigue,” and “irritability”; however, we have only used the total score in our analysis. The higher values on POMS indicate a more positive mood. In our study, the 16-item POMS total score achieved an internal consistency (reliability) across all language versions of Cronbach’s α = 0.89. Mean total scores were calculated if at least 10 items of the scale were answered by the participants.

### Statistical Analysis

Due to the hierarchically structured (i.e., participants nested in countries) and unbalanced (i.e., different numbers of participants in countries) data set, a mixed models regression approach was used for investigating the main research questions. All analyses were performed with the R software (R Core Team, 2019), and the lme4 (Bates et al., 2015) and the Ordinal (Christensen, 2019b) packages for mixed modeling.

Cumulative link mixed-models (CLMM) (Christensen, 2019a) were utilized to predict the probabilities of exercising at the five frequency levels (“never,” “1 day or less,” “2–3 days,” “4–5 days,” “almost every day”) during the pandemic by the five frequency levels of “exercise before the pandemic” (“never,” “1 day or less,” “2–3 days,” “4–5 days,” “almost every day”). The variable “country of residence” was included as a random effect.

A linear mixed model (LMM) was used to analyze the influence of exercise behavior on mood. This model was run with mood as the numerical response variable and “exercise frequency before the pandemic” and “exercise frequency during the pandemic” as categorical fixed factors (with the five levels “never,” “1 day or less,” “2–3 days,” “4–5 days,” and “almost every day”). The variable “country of residence” was included as a random effect. All fixed effects were specified with sum contrasts. Therefore, the LMM returns the grand mean dependent variable as intercept and the fixed-effect parameters as deviations from the grand mean. Pairwise *post-hoc* tests were used to compare the mood differences between all exercise frequency levels during the pandemic (e.g., never exercising before and during vs. never exercising before and 2–3 days during). Multiple testing was adjusted with the “Holm” method (model formulations, fit indices and model coefficients are provided in Supplementary Table 3).

## Results

### Exercise Behavior Change

The results show that 44.2% of the participants reported no change, 23.7% reported a decrease, and 31.9% reported an increase in their exercise frequency during the coronavirus pandemic (0.02% missing values; Supplementary Table 1 for more descriptive information).

**FIGURE 1:**
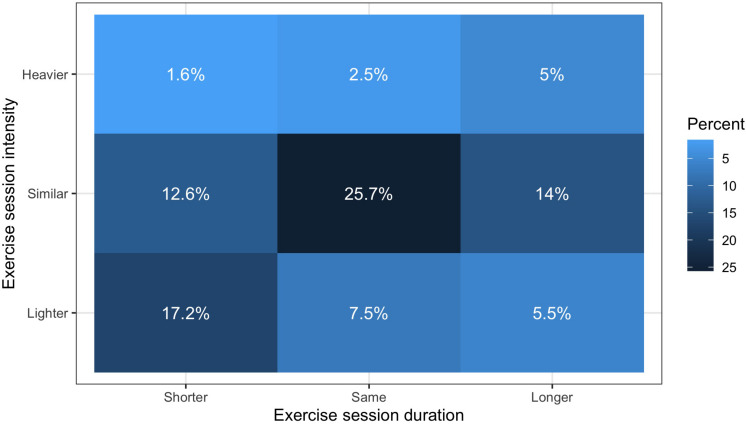
Changes in exercise behavior during the COVID-19 pandemic in March/April/May 2020 compared to prepandemic. Lighter colors show lower percentages.

Of those who exercised during the pandemic, 52.3% reported being physically active at similar, 30.2% at lower, and 9.1% at higher exercise intensities. Also, 35.7% reported the same exercise duration, 31.4% reported shorter, and 24.5% reported longer exercise duration. This information is presented in Figure 1 with more details.

A statistical model was created that can be used to predict changes in exercise frequency during similar lockdown conditions, depending on prelockdown exercise frequency. Exercise intensity and exercise duration were not included in this multilevel model because the combination of all exercise levels (“prepandemic” and “during the pandemic”) with these two ordinally ranked additional variables would have multiplied the complexity of the model and would have made statistical effects uninterpretable.

Predicting exercise frequency during lockdown conditions with prelockdown exercise frequency significantly improved the model fit compared to the null model, χ^2^_*pre*_ (4) = 3854.74, *p* < 0.001. Exercising more frequently before the pandemic significantly increased the log odds for exercising during the pandemic (*b*_*pre1*–4_ = 1.21–4.06, *p* < 0.001). Adding “country of residence” as a random effect significantly improved the model fit compared to the model without the random effect, χ^2^_*country*_ (1) = 1197.26, *p* < 0.001, supporting the rationale for using a mixed model. In the next step, “exercise before the pandemic” was added to the model as a random slope. This resulted in significant improvement in model fit, χ^2^_*pre| country*_ (14) = 108.75, *p* < 0.001, meaning that the probability of exercise frequency during the pandemic was dependent on exercise frequency before the pandemic and differed between the countries (see Supplementary Table 2 for full model specification). This indicates that potential differences in lockdown policies may have subsequently affected the changes in exercise behavior. In the fitted model, the fixed effect and random effects together explain 31.5% of variance (conditional *R*^2^) in the dependent variable. Table 2 summarizes the complete statistical results.

**TABLE 2:** Change in exercise behavior.

	Model: Exercise during the pandemic		
	
	Estimate (*SE*)	OR [95% CI]	*p*
**Location Coefficients**			
Exercise before the pandemic			
1: 1 day or less	1.21 (0.18)	3.36 [2.35–4.80]	<0.001
2: 2–3 days	1.91 (0.19)	6.75 [4.63–9.83]	<0.001
3: 4–5 days	2.71 (0.21)	15.07 [10.05–22.60]	<0.001
4: Almost every day	4.06 (0.24)	58.16 [36.38–92.98]	<0.001
**Threshold Coefficients**			
Never | 1 day or less	−0.81 (0.26)		
1 day or less | 2–3 days	0.70 (0.26)		
2–3 days | 4–5 days	2.15 (0.26)		
4–5 days | Almost every day	3.57 (0.26)		
**Random effects**			
σ^2^	3.29		
τ_00 Country_	0.88		
τ_11 Country.1 *day or less*_	0.23		
τ_11 Country.2–3 *days*_	0.30		
τ_11 Country.4–5 *days*_	0.39		
τ_11 Country. Almost every day_	0.60		
N_*Country*_	18		
Observations	13,673		
Conditional *R*^2^	0.315		

*Statistical results for the CLMM predicting exercise frequency during a lockdown with prelockdown exercise frequency.*

**FIGURE 2:**
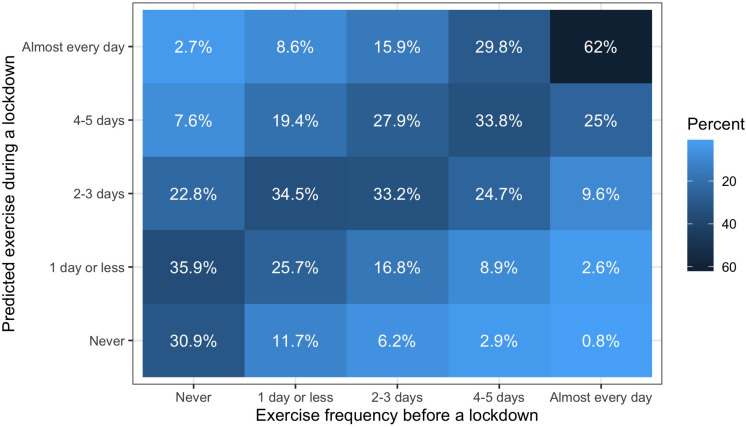
Model predictions on the probabilities of exercising during similar lockdown conditions, depending on prelockdown exercise frequency. Lighter colors indicate smaller probabilities and darker colors indicate larger probabilities. If the darkest colors were all on the diagonal from bottom left to top right, this would mean that people who exercise at a specific frequency before a lockdown would be most likely to exercise at the same frequency during a lockdown.

Figure 2 is a heatmap that illustrates the exact predictions of our model for future similar governmental lockdowns. For example, those who exercise almost every day, 4–5 days per week, and 2–3 days per week before a lockdown will most likely maintain their exercise frequency during a lockdown. Specifically, those probabilities are 62%, 33.8%, and 33.2%, respectively. However, those who exercise 1 day or less or those who never exercise before a lockdown will most likely increase their exercise frequency. Those who exercise 1 day or less will most likely (34.5%) increase their exercise frequency to 2–3 days per week, and those who are inactive before a lockdown will most likely (35.9%) increase their exercise frequency to 1 day or less per week.

### Exercise and Mood

Results of the LMM show that during the coronavirus pandemic restrictions, exercise before the pandemic (χ^2^_*pre*_ [4] = 67.38, *p* < 0.001), exercise during the pandemic (χ^2^_*during*_ [4] = 426.44, *p* < 0.001), and the interaction of these two factors (χ^2^_*pre* × *during*_ [16] = 64.14, *p* < 0.001) explained significant variability in mood during the pandemic. This means that the relationship between exercise during the pandemic and mood is different depending on how much exercise was done before the pandemic. Modeling the influence of exercise frequencies during the pandemic with random slope substantially improved model fit, χ^2^_*during| country*_ (14) = 35.38, *p* = 0.001. This means that correlations between exercise frequency during the pandemic and mood vary between countries (see Supplementary Table 3 for full model specification), and that our model controls for this variation. In the fitted model, the fixed effect and random effects together explain 8.5% of variance (conditional *R*^2^) of the dependent variable.

**FIGURE 3:**
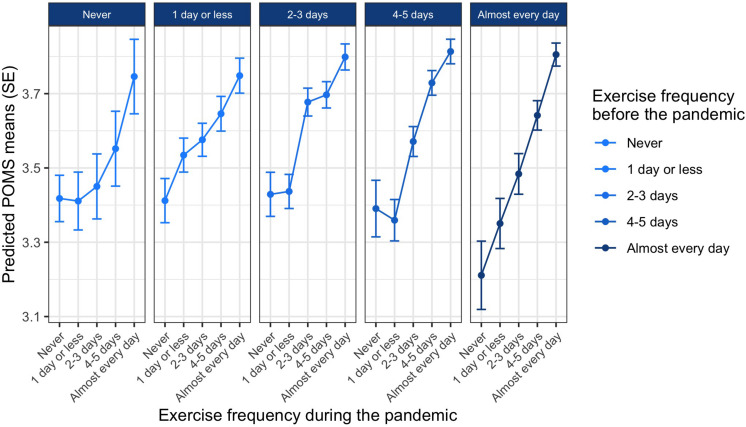
The effect of exercise frequency during the pandemic on mood depending on prepandemic exercise frequency. Lines indicate values for mood during the pandemic (higher values are better mood). Each column indicates exercise frequency before the pandemic, and exercise frequency levels within each column are exercise frequency levels during the pandemic.

Figure 3 shows the predicted values for mood at each of the five exercise frequency levels during the pandemic (within the five columns), and grouped for each exercise frequency level before the pandemic (five columns). Those who exercised almost every day during the pandemic had the best mood regardless of whether or not they exercised before the pandemic (*b*_*during4*_ = 0.23, *p* < 0.001), and there seems to be an almost linear positive correlation between exercise frequency during the pandemic and mood.

*Post-hoc* tests revealed that of those who “never” exercised, exercised “1 day or less,” or “2–3 days” before the pandemic, only those who increased their exercise frequency to “every day” during the pandemic (compared to those who maintained their pre-pandemic exercise frequency) reported significantly better mood states (*b*_*pre0:during4*–0_ = 0.33, *p* = 0.03; *b*_*pre1:during4*–1_ = 0.21, *p* = 0.001; *b*_*pre2:during4*–2_ = 0.12, *p* < 0.001). Those who reduced their exercise frequency reported worse mood states compared to those who maintained or increased their exercise frequency during the pandemic (e.g., *b*_*pre2:during1*–2_ = −0.24, *p* = 0.001; *b*_*pre3:during2*–3_ = −0.16, *p* = 0.001; *b*_*pre4:during3*–4_ = −0.16, *p* < 0.001). The only exception to the above is those who exercised “1 day or less” before the pandemic and were not physically active at all during the pandemic. Their mood did not differ from those who maintained their exercise frequency at “1 day or less” during the pandemic (*b*_*pre1:during0*–1_ = −0.12, *p* = 0.09). Results of all *post-hoc* tests are summarized in Table 3.

**TABLE 3:** *Post-hoc* tests comparing exercise levels during the coronavirus pandemic grouped by prepandemic exercise levels.

Contrast	*Estimate*	*SE*	*p*
**Pre exercise = Never (0)**			
During exercise: 1-0	–0.007	0.084	1.000
During exercise: 2–0	0.032	0.100	1.000
‘During exercise: 3–0	0.134	0.112	1.000
During exercise: 4–0	0.328	0.111	0.031
**Pre exercise = 1 day or less (1)**			
During exercise: 0–1	–0.122	0.052	0.090
During exercise: 2–1	0.041	0.047	0.385
During exercise: 3–1	0.111	0.056	0.193
During exercise: 4–1	0.214	0.056	0.001
**Pre exercise = 2–3 days (2)**			
During exercise: 0–2	–0.248	0.051	< 0.001
During exercise: 1–2	–0.241	0.041	< 0.001
During exercise: 3–2	0.020	0.028	0.964
During exercise: 4–2	0.121	0.029	< 0.001
**Pre exercise = 4–5 days (3)**			
During exercise: 0–3	–0.338	0.073	< 0.001
During exercise: 1–3	–0.370	0.056	< 0.001
During exercise: 2–3	–0.158	0.029	< 0.001
During exercise: 4–3	0.085	0.024	0.001
**Pre exercise = Almost every day (4)**			
During exercise: 0–4	–0.594	0.088	< 0.001
During exercise: 1–4	–0.455	0.0662	< 0.001
During exercise: 2–4	–0.321	0.046	< 0.001
During exercise: 3–4	–0.164	0.031	0.001

*This is a selection of comparisons showing the differences in mood for different exercise frequency levels during the pandemic when compared to maintaining the same prepandemic exercise level (for all pairwise *post-hoc* tests see Supplementary Table 4). *p* value adjustment: Holm method; Exercise levels: 0 = “Never”; 1 = “1 day or less”; 2 = “2–3 days”; 3 = “4–5 days”; 4 = “Almost every day.”*

## Discussion

This study took advantage of the lockdown rules and regulations imposed by governments from around the world during the first wave of the COVID-19 pandemic in the months of March/April/May 2020 to examine how such changes in people’s lives affect exercise behavior and SWB. We investigated mood as a component of SWB. Moreover, we have accounted for the differences between lockdown restrictions in each country which can ultimately affect exercise behavior in each nation. Using data from more than 13,000 individuals from 18 countries, we created a model that allows us to predict changes in exercise behavior during a lockdown based on prelockdown exercise behavior. According to this model we conclude that the probability of maintaining exercise frequency during a lockdown for those who exercise before a lockdown and the probability of adopting exercise for those who are inactive before a lockdown are both high. Meaning that lockdown restrictions (e.g., closing of gyms, fitness clubs, etc.) do not dramatically decrease exercise frequency for those who are frequent exercisers, and even lead to adoption of exercise for those who are inactive previous to a lockdown. We hypothesized that there will be a decrease in exercise frequency during the pandemic compared to prepandemic; however, the results show that many people maintained (44.2%), or increased (31.9%) their exercise levels, and only 23.7% reported a decrease in exercise frequency.

Interestingly, adoption of exercise or increase in exercise frequency during the pandemic for those who were inactive or rarely active before the pandemic was not associated with better mood (when compared to those who remained inactive) unless exercise frequency was drastically increased to almost every day. Importantly, those who exercised more always had better mood than those who exercised less (within the groups with different prepandemic exercise levels). Therefore, we suggest that exercising frequently during a pandemic helps with improvements in mood.

As mentioned above, we observed an almost linear dose-response relationship between exercise frequency and mood in a way that those who exercised more frequently during the pandemic also reported the most positive mood (note that in other exercise studies the dose of a particular exercise session is often more closely defined by the intensity, duration and type of exercise; this additional information was not collected in this study). Various theories, both psychological and biological, may be used to explain the connection found between exercise and mood (Landers and Arent, 2012). We consider that chronic psychological effects of exercise are more likely to explain this relationship compared to acute psychological effects as it is unlikely that acute affective responses to exercise have shaped all participants’ ratings of mood under the lockdown.

A possible psychological explanation of the effect is self-efficacy theory (Bandura, 1989). Self-efficacy is described as an individual’s belief that he/she is capable of performing a behavior with success or achieving a goal. If people see their goals realized, this can contribute to satisfaction and pride and has a positive effect on mood. Increased self-esteem and perceived physical fitness may play a role in this relation as well (Diener et al., 2017). Psychophysiological processes may also have been involved. For example, it is known that as a result of frequent exercise, there are durable changes in the activity of the hypothalamus-pituitary-adrenal axis (“stress axis”) that enable people to cope better with acute experience of stress. These changes in stress axis are associated with, for example, enhanced density and efficiency of mineralocorticoid receptors, and lower cortisol levels and the inhibition of cortisol synthesis (Matta Mello Portugal et al., 2013; see Mandolesi et al., 2018, for a review of neuroplastic phenomena as a potential mechanism involved). Physiological explanations of this kind might explain why those who decided to increase their exercise frequency only a little bit during the pandemic, did not immediately benefit from their decision with regard to SWB. However, further studies are required to shed light on the mechanisms involved.

In terms of statistical effect size, the variance in mood explained by the multilevel regression model is rather small. General mood state is indeed influenced by much more than only exercise behavior, especially during extraordinary times like the COVID-19 pandemic and the associated lockdowns in early 2020. Furthermore, it is also important to know that mood scales tend to provide score averages above the scale mean in non-clinical samples (in the POMS, with a response scale from 1 to 5, the expected mean values are usually higher than 3; Morfeld et al., 2007). Also, by taking into account that achieving maximum scores on mood scales (near a mean value of 5 in POMS) is very unusual, the exercise effect found in our study, where the mood state of those who exercise very frequently increased to near 4, may well gain some practical significance (see Figure 3). Although we do not want to exaggerate the practical importance of the mood effect identified in our data, we do believe that it is relevant for public health decision making processes during future pandemic-related lockdowns.

From the perspective of the psychological theories and results on affective responses to exercise presented in the introduction, there is a rather straightforward explanation why exercising was not associated with immediate improvements in SWB for all (Brand and Ekkekakis, 2018; Ekkekakis and Brand, 2019). It is possible that for those who are new to exercise, exercising under the lockdown restrictions was as strenuous and accompanied by the same negative acute exercise-related affect that had prevented these people from adopting an exercise routine before the pandemic. Maybe the number of exercise sessions during the lockdown was not enough to learn to enjoy exercise (i.e., to learn the cognitive appraisals necessary to transform the eventually unpleasant interoceptive signals during higher intensity exercise into a more positive affective state).

Future studies should therefore examine why people who are usually inactive or rarely active before a lockdown, tend to increase their exercise frequency during it. A speculation would be that under these circumstances (i.e., lockdown restrictions), there is an increase in boredom which can lead to an increase in need for a change and perhaps adoption of a new behavior such as exercise. Importantly, future research should investigate whether human beings, if given a chance, have a tendency to become physically active (Pontzer et al., 2012), or on the other hand have a tendency to minimize their physical activity and save energy (Cheval et al., 2018). Increase in physical activity under the pandemic restrictions as seen in our data, might be a sign of potential predisposition to physical activity in human beings that was less likely to occur under the conditions of daily life routines before the restrictions (e.g., work, family, and personal interests).

Our data also suggests that about one third of the participants lowered exercise intensities (30.2%) and shortened exercise durations (31.4%) during the lockdown (24.5% of the participants reported to have increased session durations). Because our survey focused primarily on the changes in exercise frequency (we considered it more important to learn whether exercise was done at all), we are unfortunately not able to comment on the changes in exercise type that occurred as the consequence of the lockdown. Such phenomena should be analyzed and additional information should be collected with follow-up studies.

Among the strengths of this study is that it was initiated and conducted during the brief and unexpected period of time of the governmental lockdown restrictions related to the first wave of the 2020 coronavirus pandemic, using this difficult time as an opportunity to investigate changes in exercise behavior and their effects on mood on a very large scale. To overcome language barrier, the questionnaire was translated into 18 languages which made it possible for many non-English speakers across the globe to participate. In fact, we were able to reach more than 13,000 participants in a relatively short period of time (i.e., a month). The large sample size allowed us to adjust for national differences in our analysis, which accounts for both lockdown rules and regulations and cultural differences. Therefore, we can generalize the results to 18 countries included in the statistical modeling (see Supplementary Table 3 for full model specification) knowing that different rules and regulations and cultural differences would not impact our results.

In order to collect data from a very large sample of participants all over the world during the limited time of governmental lockdowns, some compromises had to be made and therefore, limitations are present in this study. The study was cross-sectional and the data on exercise behavior was collected by self-report which might be subject to bias and poor memory recall. This may have particularly affected our measurement of prepandemic exercise frequency. Study participants are often inclined to overstate their own exercise levels in surveys (Brenner and DeLamater, 2014). The retrospective assessment of prepandemic exercise behavior may have increased this bias. Furthermore, because we did not ask participants about their current health status (except for a possible infection with COVID-19), some participants may have reduced their exercise frequency during the pandemic not because of the lockdown but because of other diseases or injuries. We also refrained from using a standardized, yet extensive physical activity questionnaire such as IPAQ (Hagströmer et al., 2006). Instead, we intended to avoid boredom and fatigue for the participants by presenting a very brief instrument, and using different logics to streamline the flow. Another limitation is that the nature of online data collection might limit the sample to certain groups (e.g., younger, tech-savvy/technophile); however, the demographic information of the participants shows a rather heterogeneous sample and an average age of 34 years (*SD* = 14.4 years) (Table 3, “Materials and Methods”).

This study has several implications. First, it investigated mood as an important aspect of SWB and the results show that exercising during a lockdown contributes to positive SWB. This contributes to basic research in psychology investigating behavioral factors associated with SWB (Diener et al., 2017). Interventionists may feel encouraged to examine how SWB could be targeted during times of crisis specially for those who have always found exercise unattractive as a lifestyle element or those who live with a disability. Social marketing approaches may be particularly relevant here, because health behavior change messages in times of lockdowns would probably have to be communicated primarily through the media (Evans, 2006). Second, we created a model that predicts changes in exercise frequency during a lockdown based on prelockdown exercise frequency. These results could be of interest to behavioral researchers as they can use this model to elaborate on public health strategies for upcoming times with similar lockdown restrictions. Third, our results indicate that those who were inactive during a lockdown, had worse SWB compared to others. This is important as dampened mood states are associated with less self-control which in itself is shown to be an important determinant of complying with restrictive rules such as social distancing (Martarelli and Wolff, 2020; Wolff et al., 2020). Therefore, policy makers can use these results and promote exercise and physical activity in their countries to be able to benefit from its positive effects on mood under similar lockdown restrictions in the future. In fact, it is not entirely implausible that exercising in good times can help people get through the hard times easier (Silverman and Deuster, 2014).

## Conclusion

This study investigated the changes in exercise behavior and the respective changes in SWB during the coronavirus pandemic lockdown restrictions in early 2020. The results show that the lockdown restrictions did not lead to decrease in exercise levels. Also, those who exercised more frequently during the pandemic reported the most positive mood states. Our prediction model (Figure 2) can inform policy makers how exercise behavior may change again during future epidemics with governmental restrictions or lockdowns. Interestingly, early positive effects on well-being for those who normally avoid exercise are likely to occur only if they try to exercise almost every day. These results contribute to basic research in psychology and may be of interest to behavioral researchers and interventionists as well.

## Data Availability Statement

The dataset used in this study is publicly available in the online repository here: https://osf.io/qh6et/.

## Ethics Statement

Ethical review and approval was not required for the study on human participants in accordance with the local legislation and institutional requirements. The patients/participants provided their written informed consent to participate in this study.

## Author Contributions

All authors listed have made a substantial, direct and intellectual contribution to the work, and approved it for publication.

## Conflict of Interest

The authors declare that the research was conducted in the absence of any commercial or financial relationships that could be construed as a potential conflict of interest.
