# Supplementary material for: When Pandemic Hits: Exercise Frequency and Subjective Well-Being During COVID-19 Pandemic
Source: Front Psychol. 2020 Sep 24;11:570567. doi: 10.3389/fpsyg.2020.570567 (PMC7541696; doi:10.3389/fpsyg.2020.570567)
Supplement: Supplementary file 1 [file Table_1.DOCX]

**Additional File 1
Supplementary Tables**

**When pandemic hits:
Exercise frequency and subjective well-being
during COVID-19 pandemic**

Authors:

1. *Ralf Brand, University of Potsdam, Germany (ralf.brand@uni-potsdam.de)
2. Sinika Timme, University of Potsdam, Germany
3. Sanaz Nosrat, Lehman College New York, USA

**Supplementary Table 1.** Exercise frequency before and during the pandemic for participants who met the inclusion criteria.

| **Exercise before** | **Exercise during** | ***n*** |
| --- | --- | --- |
| Never | Never | 201 |
| Never | 1 day or less | 86 |
| Never | 2-3 days | 61 |
| Never | 4-5 days | 44 |
| Never | Almost every day | 42 |
| 1 day or less | Never | 258 |
| 1 day or less | 1 day or less | 728 |
| 1 day or less | 2-3 days | 482 |
| 1 day or less | 4-5 days | 300 |
| 1 day or less | Almost every day | 272 |
| 2-3 days | Never | 269 |
| 2-3 days | 1 day or less | 604 |
| 2-3 days | 2-3 days | 1545 |
| 2-3 days | 4-5 days | 1020 |
| 2-3 days | Almost every day | 858 |
| 4-5 days | Never | 106 |
| 4-5 days | 1 day or less | 230 |
| 4-5 days | 2-3 days | 823 |
| 4-5 days | 4-5 days | 1642 |
| 4-5 days | Almost every day | 1208 |
| Almost every day | Never | 64 |
| Almost every day | 1 day or less | 131 |
| Almost every day | 2-3 days | 210 |
| Almost every day | 4-5 days | 550 |
| Almost every day | Almost every day | 1939 |
| NA | NA | 23 |
| **Total** |  | **13,696** |

| **Supplementary Table 2.** Model selection and fit indices for predicting exercise behaviour during a lockdown with exercise behaviour before a lockdown. (Predictions for future similar lockdown conditions) |
| --- |

| **Model** | **nobs** | **npar** | **AIC** | **logLik** | **test** | **LR.stat** | **df** | ***p*** |
| --- | --- | --- | --- | --- | --- | --- | --- | --- |
| Ex_during ~ 1 | 13673 | 4 | 40908 | -20450 |  |  |  |  |
| Ex_during ~ Ex_before | 13673 | 8 | 37062 | -18523 | 1 vs 2 | 3854.74 | 4 | <.001 |
| Ex_during ~ Ex_before + (1\|country) | 13673 | 9 | 35866 | -17924 | 2 vs 3 | 1197.26 | 1 | <.001 |
| Ex_during ~ Ex_before + (Ex_before \|country) | 13673 | 23 | 35786 | -17870 | 3 vs 4 | 108.75 | 14 | <.001 |

nobs: number of observations
npar: number of parameters

| **Supplementary Table 3.** Model selection and fit indices for predicting mood with exercise behaviour during and before the coronavirus pandemic. |
| --- |

| **Model** | **nobs** | **npar** | **AIC** | **BIC** | **logLik** | **deviance** | **test** | **Chisq** | **df** | ***p*** |
| --- | --- | --- | --- | --- | --- | --- | --- | --- | --- | --- |
| POMS *~* 1 + (1\|country) | 13500 | 3 | 26210 | 26232 | -13102 | 26204 |  |  |  |  |
| POMS ~ Ex_before+ (1\|country) | 13500 | 7 | 26150 | 26203 | -13068 | 26136 | 1 vs 2 | 67.38 | 4 | <.001 |
| POMS ~ Ex_during+ (1\|country) | 13500 | 7 | 25791 | 25844 | -12889 | 25777 | 1 vs 2 | 426.44 | 4 | <.001 |
| POMS ~ Ex_before + Ex_during + (1\|country) | 13500 | 11 | 25786 | 25868 | -12882 | 25764 | 2 vs 4 | 372.75 | 4 | <.001 |
| POMS ~ Ex_during * Ex_before + (1\|country) | 13500 | 27 | 25754 | 25956 | -12850 | 25700 | 4 vs 5 | 64.14 | 16 | <.001 |
| POMS ~ Ex_during * Ex_before + (Ex_before \|country) | 13500 | 41 | 25771 | 26079 | -12845 | 25689 | 5 vs 6 | 10.42 | 14 | 0.731 |
| POMS ~ Ex_during * Ex_before + (Ex_during \|country) | 13500 | 41 | 25746 | 26054 | -12832 | 25664 | 5 vs 7 | 35.38 | 14 | =.001 |

nobs: number of observations
npar: number of parameters

| **Supplementary Table 4.** Pairwise post hoc tests comparing exercise levels during the coronavirus pandemic for pre exercise groups. |
| --- |

| **Contrast** | **Estimate** | ***SE*** | ***p*** |
| --- | --- | --- | --- |
| Pre exercise = Never (0) |  |  |  |
| During exercise: 0-1 | 0.007 | 0.084 | 1.000 |
| During exercise: 0-2 | -0.032 | 0.100 | 1.000 |
| During exercise: 0-3 | -0.134 | 0.112 | 1.000 |
| During exercise: 0-4 | -0.328 | 0.111 | 0.031 |
| During exercise: 1-2 | -0.039 | 0.109 | 1.000 |
| During exercise: 1-3 | -0.141 | 0.123 | 1.000 |
| During exercise: 1-4 | -0.335 | 0.122 | 0.054 |
| During exercise: 2-3 | -0.102 | 0.126 | 1.000 |
| During exercise: 2-4 | -0.296 | 0.126 | 0.151 |
| During exercise: 3-4 | -0.194 | 0.136 | 1.000 |
| Pre exercise = 1 day or less (1) |  |  |  |
| During exercise: 0-1 | -0.122 | 0.052 | 0.090 |
| During exercise: 0-2 | -0.164 | 0.056 | 0.020 |
| During exercise: 0-3 | -0.234 | 0.064 | 0.002 |
| During exercise: 0-4 | -0.336 | 0.064 | <.001 |
| During exercise: 1-2 | -0.041 | 0.047 | 0.385 |
| During exercise: 1-3 | -0.111 | 0.056 | 0.193 |
| During exercise: 1-4 | -0.214 | 0.056 | 0.001 |
| During exercise: 2-3 | -0.070 | 0.048 | 0.280 |
| During exercise: 2-4 | -0.173 | 0.049 | 0.003 |
| During exercise: 3-4 | -0.103 | 0.053 | 0.193 |
| Pre exercise = 2-3 days (2) |  |  |  |
| During exercise: 0-1 | -0.008 | 0.053 | 0.964 |
| During exercise: 0-2 | -0.248 | 0.051 | <.001 |
| During exercise: 0-3 | -0.268 | 0.057 | <.001 |
| During exercise: 0-4 | -0.370 | 0.056 | <.001 |
| During exercise: 1-2 | -0.241 | 0.041 | <.001 |
| During exercise: 1-3 | -0.260 | 0.048 | <.001 |
| During exercise: 1-4 | -0.362 | 0.046 | <.001 |
| During exercise: 2-3 | -0.020 | 0.028 | 0.964 |
| During exercise: 2-4 | -0.121 | 0.029 | <.001 |
| During exercise: 3-4 | -0.102 | 0.029 | 0.002 |
| Pre exercise = 4-5 days (3) |  |  |  |
| During exercise: 0-1 | 0.031 | 0.078 | 0.686 |
| During exercise: 0-2 | -0.181 | 0.071 | 0.022 |
| During exercise: 0-3 | -0.338 | 0.073 | <.001 |
| During exercise: 0-4 | -0.423 | 0.072 | <.001 |
| During exercise: 1-2 | -0.212 | 0.054 | <.001 |
| During exercise: 1-3 | -0.370 | 0.056 | <.001 |
| During exercise: 1-4 | -0.454 | 0.055 | <.001 |
| During exercise: 2-3 | -0.158 | 0.029 | <.001 |
| During exercise: 2-4 | -0.242 | 0.030 | <.001 |
| During exercise: 3-4 | -0.085 | 0.024 | 0.001 |
| Pre exercise = Almost every day (4) |  |  |  |
| During exercise: 0-1 | -0.139 | 0.100 | 0.164 |
| During exercise: 0-2 | -0.273 | 0.095 | 0.012 |
| During exercise: 0-3 | -0.430 | 0.092 | <.001 |
| During exercise: 0:4 | -0.594 | 0.088 | <.001 |
| During exercise: 1-2 | -0.133 | 0.076 | 0.157 |
| During exercise: 1-3 | -0.291 | 0.071 | <.001 |
| During exercise: 1-4 | -0.455 | 0.066 | <.001 |
| During exercise: 2-3 | -0.158 | 0.052 | 0.009 |
| During exercise: 2-4 | -0.321 | 0.046 | <.001 |
| During exercise: 3-4 | -0.164 | 0.031 | <.001 |

*Note*. *p* value adjustment: Holm method

Exercise levels: 0 = “Never”; 1 = “1 day or less”; 2 = “2-3 days”; 3 = “4-5 days”; 4 = “Almost every day”
